# Unusual Presentation of Recurrent Calcinosis Cutis in the Right Thumb of a 16-Year-Old Female

**DOI:** 10.7759/cureus.59721

**Published:** 2024-05-06

**Authors:** Munir Alam, Abdullah S ALAhmari, Ahmed S ALAhmari

**Affiliations:** 1 Plastic Surgery, King Abdullah Hospital Bisha, Bisha, SAU; 2 Orthopedics, Armed Forces Hospital Southern Region, Khamis Mushait, SAU; 3 Medicine and Surgery, Bisha University, Bisha, SAU

**Keywords:** idiopathic, multifactorial, calcium deposition, recurrent, calcinosis cutis

## Abstract

Calcinosis cutis is a quite unusual disease represented by abnormal accumulation of calcium salts in the skin and subcutaneous tissues. Repeated cutis calcinosis means recurrent calcium deposition in pre-existing areas. The case report illustrated the case of a 16-year-old female who had recurrent calcium deposits on the base of her right thumb. The patient initially had swelling at the base of her right thumb, which had been present for six months now. The patient described the dorsal solid mass on top of the thumb base, which was painful and had reduced thumb mobility. There was swelling that became painful, specifically located at the same site as the previous surgery, with thumb restriction and superadded infection at the metacarpophalangeal joint. Routine lab tests, including blood tests and rheumatologic and autoimmune work-ups, were normal. Plain radiographs and ultrasound examinations unveiled the characteristics of calcifications in the thumb tissues. A skin biopsy was done and the calcium deposits in subcutaneous tissue were confirmed, matching calcinosis cutis. The approach to the treatment of this condition entailed conservative measures. Some included physiotherapy to correct a flexion deformity, antibiotics, painkillers, and daily dressing. The patient was advised to follow up and to consider excision of the nodules. This case points out the clinical manifestations, investigations, and initial management of available strategies for recurrent calcinosis cutis. Further studies and long-term follow-up are necessary to determine the optimal treatment approaches and outcomes for this rare condition.

## Introduction

Calcinosis cutis is an uncommon syndrome that presents with the abnormal deposition of calcium salts in the skin and subcutaneous tissues. In simple words, recurrent calcinosis cutis is the chronic deposition of calcium salts under the skin in different areas. This phenomenon, although rare, holds serious consequences for patients, thus demanding much attention [1]. In this case report, we present the case of a 16-year-old girl who had recurrent problems of calcinosis cutis situated at the base of her right thumb.

While calcinosis cutis represents a rare condition and its exact incidence is yet to be determined, calcinosis universalis manifests itself with solid, chalky nodules involving the subcutaneous and fibrous structures of the muscles and tendons [2]. Moreover, recurrent calcinosis cutis is extremely rare and, as a result, only a few data on its incidence can be found in the studies conducted on this health disorder. In addition to this, the rare presentation of the challenges of recurrent calcinosis cutis in the thumb further adds to the uniqueness of this case.

The etiology of calcinosis cutis is multifactorial, with many underlying diseases added as potential reasons. They present as connective tissue, autoimmune, and metabolic disorders, trauma, and genetic predisposition [3]. Nevertheless, in this specific situation, the absence of systemic manifestations and negative results in the rheumatology and autoimmune workup indicates that the patient has an idiopathic form of calcinosis cutis.

The diagnostics of recurrent calcinosis cutis can be a difficult issue given its rarity and the variances of the manifestations. The role of imaging in visualizing the calcifications in the affected tissues is crucial, and plain radiograph screening and ultrasound are critical in this context. Other kinds of diagnostic approaches, such as laboratory tests and skin biopsies, become necessary where the presence of calcium deposits has been confirmed [4].

The lack of management recommendations for recurrent cutaneous calcinosis is striking as there is still no consensus on best practices and evidence-based guidelines. Treatment is normally conservative in nature and directed toward symptomatic relief, which involves giving physiotherapy to prevent or manage functional decline, treat the pain, and provide wound care. Depending on the circumstances, excision surgery of bone areas with tiny deformations might be used to reduce the level of pain and to improve the functions [5].

In this case report, we augment existing medical literature and highlight the clinical manifestations, diagnostic evaluation, and initial management strategies for recurrent calcinosis cutis. However, further research and long-term follow-up are needed to better understand the pathogenesis, optimize treatment strategies, and assess the long-term outcomes in patients with this rare condition.

## Case presentation

**Figure 1:**
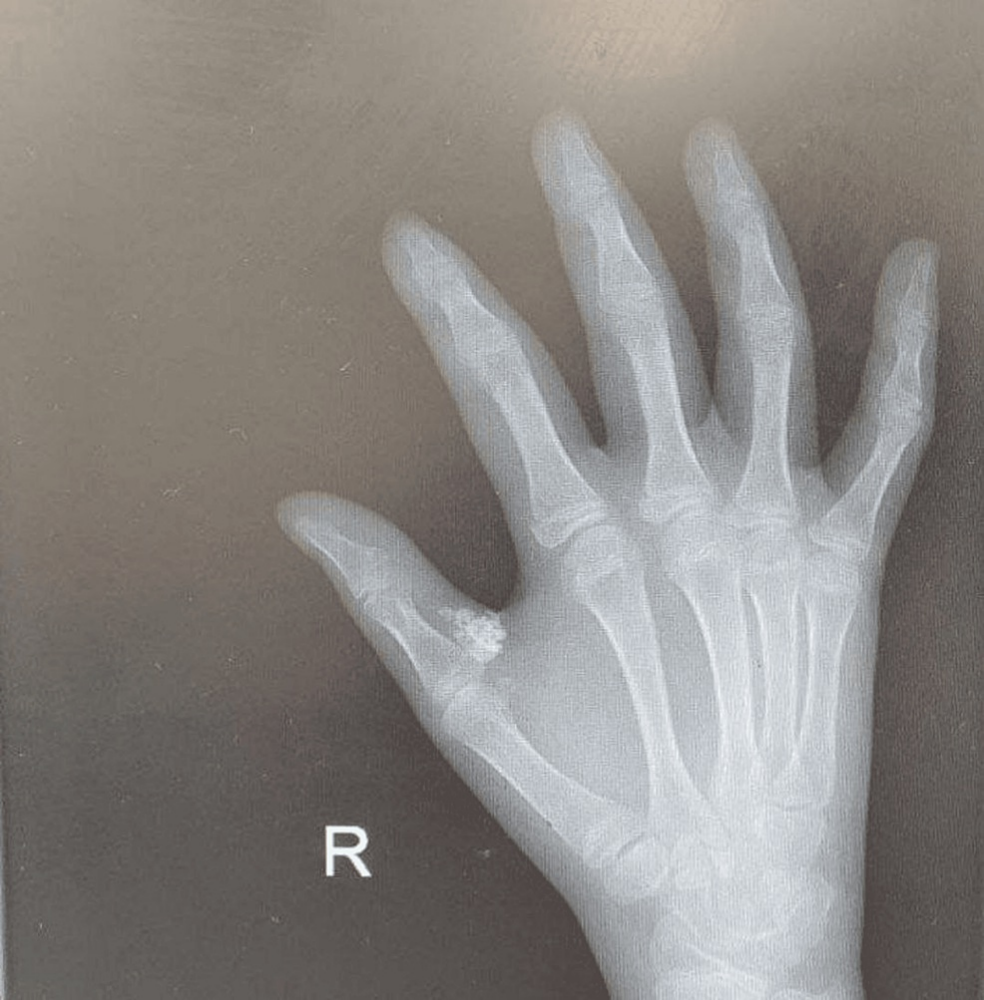
Plain X-ray of the recurrent calcinosis cutis specifically affecting her right thumb's metacarpophalangeal joint

A 16-year-old girl came to the hospital with a quantity of growth occurring under the base of her right thumb quite specifically. During that process, the patient has stated a gradual onset of symptoms over the course of the last six months. First, she noted a palpable nodule of small and firm size located on the dorsal aspect of the right thumb, which slowly started to enlarge and become more painful. Along with other crop-outs and plaques that grew in the place (Figure 1), it led to a significant unpleasant feeling and difficulty of the thumb to move.

**Figure 2:**
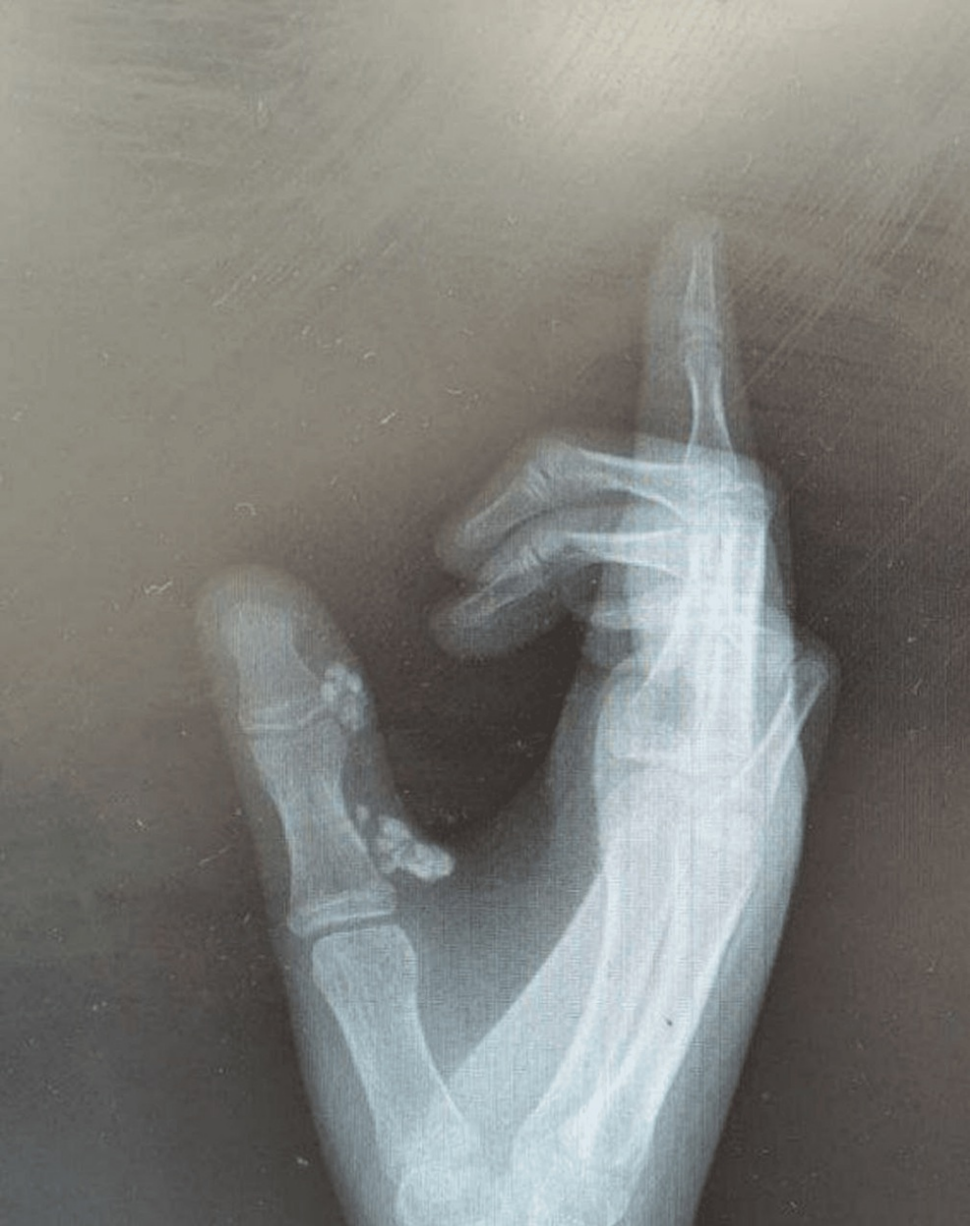
Plain X-ray of the calcinosis cutis specifically affecting her right thumb's interphalangeal joint and metacarpophalangeal joint

The patient exhibited a scan of the previous history of the lesions in the same area of the base of the thumb of the right hand that had been excised and biopsied under general anesthetic about two years ago as shown in Figure 2. Histological examinations confirmed that there was classical calcinosis cutis.

There were no similar lesions found in the breast, genitalia, or any other part of the body. Also, the lesion was non-traumatic, and no history of pathologic lesions was seen at the site of the nodule. Moreover, there was no significant family history of chronic diseases, and those conditions were not reported.

**Figure 3:**
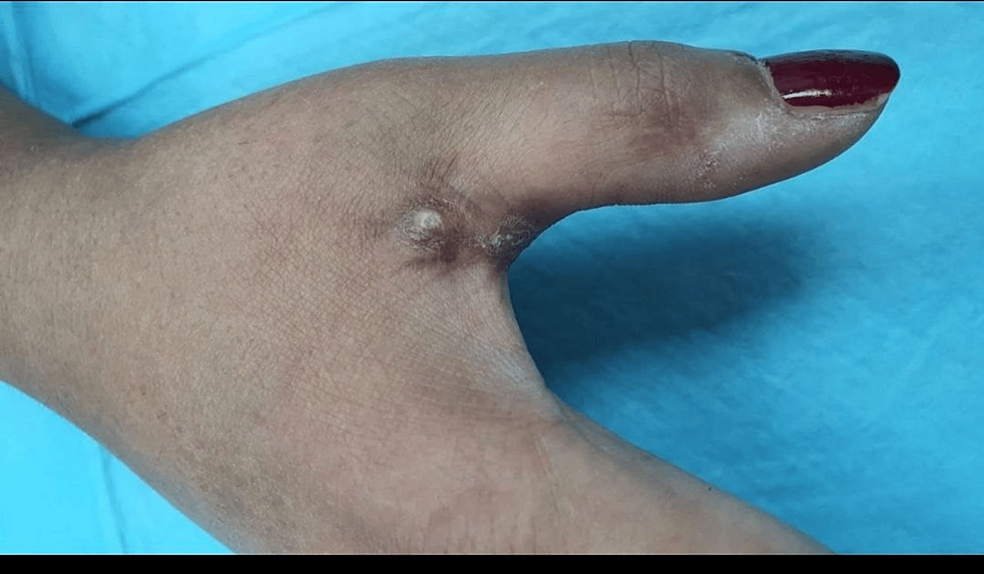
Clinical photograph demonstrating nodularity in the right thumb's metacarpophalangeal joint

Physical examination revealed several well-defined, firm nodules measuring approximately 1.5x1x0.7 cm in size. These nodules were principally found in the mid-visual area of the right thumb metacarpophalangeal joint (Figure 3). There was redness and swelling in the same area as the prior surgery, with thumb limitation and cellulitis at the base of the second operation. Similarly, in the previous calculation, there was calcinosis cutis over the right thumb’s metacarpophalangeal joint and interphalangeal joint and this is demonstrated in Figure 2. At the right thumb, there are two lesions present, one in the interphalangeal joint and the other in the metacarpophalangeal joint, The thumb provides 50% of hand function. On examination at three-month postoperative intervals, this patient had regained her normal movements. She was also feeling normal sensations with a good tactile feeling, and there was no numbness or tingling when touched lightly or pricked.

**Figure 4:**
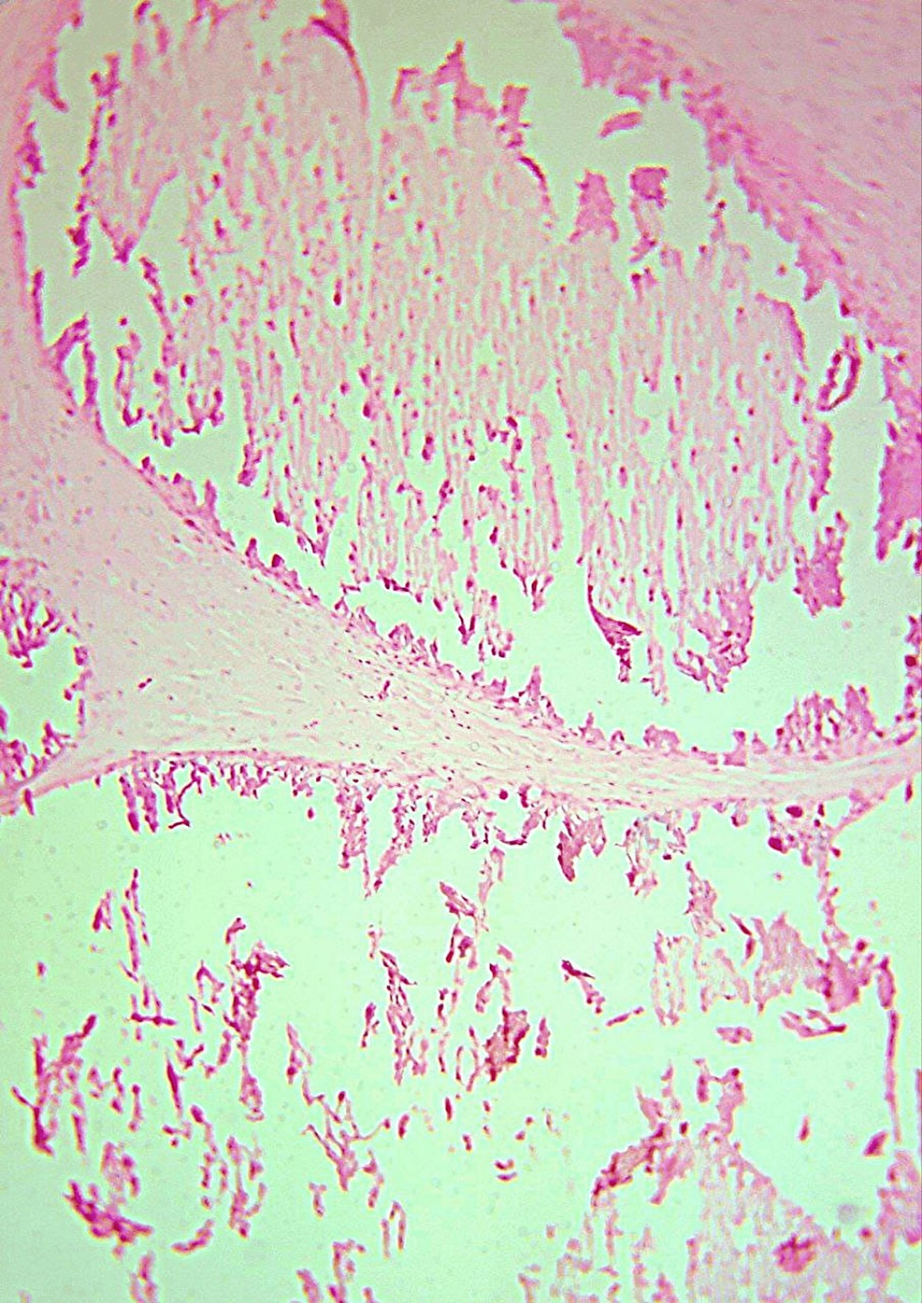
A high-power field histopathological image shows skin tissue exhibiting irregular deposits of intensely basophilic acellular material in the dermis and subcutaneous tissue The deposits are typically well-circumscribed with a thin rim of eosinophilic hyalinisation. No atypia or malignancy was seen.

Laboratory explorations, including blood tests, demonstrated normal outcomes from the assessment of CBC, renal and hepatic function tests, and serum calcium and phosphate levels. Workup for rheumatologic and autoimmune conditions was performed with negative results for antinuclear antibody (ANA) and rheumatoid factor (RF). The Endocrinology workup resulted negative without any concerning endocrinologic reason that could be associated with this case. Radiographic modality, which includes plain radiographs and ultrasound, detected classic calcifications within the soft tissues of the crippled thumb. The skin biopsy showed calcium deposits in the subcutaneous tissue, which corresponded with calcinosis cutis (Figure 4).

The treatment approach involved a conservative management plan. Physiotherapy was recommended to address the flexion deformity, and the patient received oral clindamycin 300 mg 1-1-1×10 days for suspected Methicillin-resistant Staphylococcus aureus (MRSA) infection, along with fusidate sodium skin ointment and non-steroidal anti-inflammatory drugs 1-0-1×10 days for pain relief. Daily dressings were performed to manage the affected area. The patient was advised to follow up and consider excision of the nodules. Physiotherapy was recommended during the postoperative recovery phase to restore the movement. During the first week, the range of motion was not changed after surgical intervention. Gradually, it started getting better with physiotherapy, and after a month, the range of motion was within normal limits.

Given the diagnosis of recurrent calcinosis cutis affecting the right thumb, a multidisciplinary approach was adopted to manage the patient's condition.

## Discussion

Calcinosis cutis encompasses five subtypes: dystrophic, metastatic, idiopathic, iatrogenic calcification, and calciphylaxis [2]. Dystrophic calcification arises from local tissue damage or abnormalities, such as connective tissue disorders, and is associated with normal calcium and phosphate levels. Metastatic calcification results from abnormal calcium and/or phosphate metabolism, leading to calcium deposition in tissues. Iatrogenic calcinosis occurs as a complication of intravenous administration of calcium or phosphate. Calciphylaxis manifests as a calcifying vasculopathy affecting small vessels [3].

In our case discussion, we excluded other types of calcinosis cutis to arrive at the final diagnosis. Calciphylaxis, metastasis, and tumoral calcinosis cutis were ruled out due to normal calcium and phosphate levels. Dystrophic calcinosis cutis was unexpected based on normal immunological tests and the absence of clinical symptoms [4].

When we have already excluded these types, we are left with only one type of idiopathic calcinosis cutis that is caused by no underlying metabolic disorder or tissue injury. Similarly, this subtype is varied and includes nodules that reside in subepidermal, calcified tissues, and scrotal calcinosis. In our patient, we are being faced with the very uncommon case of the recurrence of calcinosis in the right thumb in a 16-year-old girl, which is one of the cases that are difficult to diagnose and treat. There was a widespread manifestation of idiopathic calcinosis cutis, with the exact etiology remaining unknown.

 The treatment approach for the recurrent calcinosis cutis in the right thumb initially involved conservative management, including oral clindamycin, for suspected MRSA infection, along with topical fusidate sodium ointment and non-steroidal anti-inflammatory drugs for pain relief. Alternate day dressings were performed, and physiotherapy was recommended to address deformity limitations [6]. However, it improved through physiotherapy during this period, which was almost a month long. Sensation and motor systems were not affected. The resilience to medical treatment may continue to progress over time, potentially leading to further functional limitations, cosmetic concerns, and decreased quality of life for the patient. As a result, the surgical removal of this lesion avoids the development of more complex disease complications and leads to better treatment effects and the patient's healthy life.

To avoid recurrence, we have made comprehensive postoperative care a priority. It includes regular patient check-ups, abiding by wound-care guidelines, teaching the patient how to maintain thumb cleanliness, and avoiding activities that can worsen the condition. Physiotherapy including olive oil massage is the key element to rehabilitation improvement of thumb function and prevention of mobility problems. Follow-up examinations with healthcare providers should be closely observed, as it is an important way to detect recurrence and complications and ensure a good outcome.

It should be recognized that the management of recurrent calcinosis cutis could be difficult because of the rarity of this condition and the insufficiency of evidence-based guidelines. Each case must be individually evaluated and includes a multidisciplinary approach with consideration of clinical presentation, the particularities of a patient, and expected treatment outcomes.

## Conclusions

This case report focused on a distinct manifestation of calcinosis cutis recurrence in a 16-year-old girl, which primarily involved her right thumb. The management of this condition is challenging, which is mainly due to its unique location and recurrent nature. Conservative measures may be first introduced, but in situations where the response is minimal or the functional outcome is severely affected, surgical excision is considered. We stress the importance of long-term follow-up, which entails tracking for relapse and optimal outcomes. This case adds to the ongoing medical literature in a way that underscores the role of early detection, accurate diagnosis, and particular management plans for recurrent calcinosis cutis.

This rarity of the disease necessitates that further research might be needed to understand the pathophysiology of this disorder and stick to the standardized guidelines for the diagnosis and management of this disease. However, it is worth noticing that it is just one case history of a particular patient, and the results may not be similar in all cases of recurrent calcinosis cutis. Each patient with the disease has a specific requirement for treatment that is dependent on some basic factors such as the severity of the disease and the expected response to the available treatment.
